# Green Bioanalytical Applications of Graphene Oxide for the Extraction of Small Organic Molecules

**DOI:** 10.3390/molecules26092790

**Published:** 2021-05-09

**Authors:** Natalia Manousi, Orfeas-Evangelos Plastiras, Eleni A. Deliyanni, George A. Zachariadis

**Affiliations:** 1 Laboratory of Analytical Chemistry, Department of Chemistry, Aristotle University of Thessaloniki, 54124 Thessaloniki, Greece; orfeplas@hotmail.com; 2 Laboratory of Chemical and Environmental Technology, Department of Chemistry, Aristotle University of Thessaloniki, 54124 Thessaloniki, Greece; lenadj@chem.auth.gr

**Keywords:** bioanalysis, graphene oxide, sample preparation, blood, urine, small organic molecules

## Abstract

Bioanalysis is the scientific field of the quantitative determination of xenobiotics (e.g., drugs and their metabolites) and biotics (e.g., macromolecules) in biological matrices. The most common samples in bioanalysis include blood (i.e., serum, plasma and whole blood) and urine. However, the analysis of alternative biosamples, such as hair and nails are gaining more and more attention. The main limitations for the determination of small organic compounds in biological samples is their low concentration in these matrices, in combination with the sample complexity. Therefore, a sample preparation/analyte preconcentration step is typically required. Currently, the development of novel microextraction and miniaturized extraction techniques, as well as novel adsorbents for the analysis of biosamples, in compliance with the requirements of Green Analytical Chemistry, is in the forefront of research in analytical chemistry. Graphene oxide (GO) is undoubtedly a powerful adsorbent for sample preparation that has been successfully coupled with a plethora of green extraction techniques. GO is composed of carbon atoms in a sp^2^ single-atom layer of a hybrid connection, and it exhibits high surface area, as well as good mechanical and thermal stability. In this review, we aim to discuss the applications of GO and functionalized GO derivatives in microextraction and miniaturized extraction techniques for the determination of small organic molecules in biological samples.

## 1. Introduction

Bioanalysis is the sub-discipline of analytical chemistry that deals with the quantitative determination of the exogenous and endogenous chemical compounds in biological matrices. Exogenous compounds are known as xenobiotics (e.g., drugs and their metabolites), while the endogenous ones are called biotics (e.g., macromolecules and biomarkers). Based on the analytical purpose, two distinct types of methodologies can be followed in bioanalysis: untargeted and targeted analysis. Targeted analysis is applied to determine a specific analyte or analytes, while untargeted analysis is mainly applied to obtain a maximum of information from the sample in order to isolate inter- or intra-individual variations and identify potential biomarkers [1,2]. Unequivocally, bioanalysis is a significant tool for many scientific fields, since it provides significant information regarding the absorption, distribution, metabolism and elimination of drugs [1].

A classical bioanalytical procedure is normally composed of two parts; the sample preparation and the detection/quantification of the target analyte [3,4,5]. Generally, the analysis of xenobiotics in biological matrices is a challenging procedure due to their low concentration, in combination with the complexity of the samples. Among the conventional samples that are examined for bioanalytical methods are blood serum, blood plasma, whole blood and urine while in the recent years, the analysis of alternative matrices (e.g., saliva, hair, nails, sweat and cerebrospinal fluid) has also gained attention [6]. Biological samples and pharmaceutical products are complex matrices that often contain macromolecules, inorganic salts and organic compounds that might exhibit similar chemical or physical properties with the target analytes. As a result, a clean-up and a preconcentration step are typically required [7,8].

Solid-phase extraction (SPE), liquid-liquid extraction (LLE), protein precipitation and direct injection are the four major techniques that have been employed for the sample preparation of biological matrices [9]. However, both SPE and LLE suffer from some fundamental limitations including the requirement for high sample quantities and high consumption of hazardous organic solvents [8,10]. Since the introduction of Green Analytical Chemistry (GAC) that emerged from Green Chemistry, continuous effort is being made to develop environmental friendly analytical methodologies as sustainable alternatives to classical sample preparation methods [11]. As a result, a plethora of microextraction and miniaturized techniques, such as solid-phase microextraction (SPME) [12], liquid phase microextraction (LPME) [13], dispersive solid phase extraction (d-SPE) [14,15,16], magnetic solid-phase extraction (MSPE) [17], stir bar sorptive extraction (SBSE) [18], pipette tip solid-phase extraction (PT-SPE) [19] and fabric phase sorptive extraction (FPSE) [20] have arisen. Compared to the conventional sample preparation approaches, microextraction and miniaturized extraction techniques require the consumption of lower sample and organic solvent quantity and thus they are considered more environmentally friendly [11].

At the same time, the discovery of new materials has also attracted the interest of many researchers working in the field of sample preparation [21]. Among them, carbon-based extraction sorbents, such as fullerenes, carbon nanotubes, graphene and graphene oxide (GO) have been extensively studied in extraction techniques, due to their benefits including high thermal and mechanical stability, as well as high surface area [22,23,24]. Graphene is a 2-D carbon-based material that presents a hexagonal structure made from sp^2^ hybridized carbon atoms [25]. Graphene is considered to be a promising tool for the extraction of benzenoid form compounds due to its large delocalized π-electron system that is able to form strong π-stacking interactions with the π-electron rich benzene rings of small organic molecules [23]. GO is a chemical compound that exhibits a relatively polar and hydrophilic character, since it contains a large number of oxygen-containing groups including hydroxyl, carboxyl, and epoxy groups [26]. As a result, GO is usually applied for the extraction of polar and hydrophilic compounds through electrostatic interactions, dative bonds, hydrogen bonds and cation-π interactions, while graphene is typically used to adsorb non-polar organic compounds with aromatic rings through π–stacking interactions [27,28]. Finally, it should be noted that functionalization of graphene can enhance the selectivity of the sorbent towards the target analytes [26]. Until now, GO has been coupled to various green sample preparation techniques, while it is also used in the development of biosensors [29,30].

Undoubtedly, GO is a powerful adsorbent that has gained a lot of attention in sample preparation. As a result, numerous reviews have been published discussing its application in analytical chemistry [22,25,26,31,32,33]. This review discusses the applications of GO and its functionalized derivatives in green sample preparation techniques for the determination of small organic molecules in biological samples. Attention is paid to the applications of miniaturized extraction techniques and microextraction techniques that were reported in the literature during the last 5 years.

## 2. Preparation of GO and GO Derived Materials

The first time graphene and graphite oxide were prepared was reported in literature in 1957 by Hummers [34]. Since then, many modifications have been made that require less reagents and are more eco-friendly.

Alkhouzaam et al. [35] tried two different approaches in the synthesis of GO by tweaking the weight of the reagents used, as well as the temperature of the reaction and the time of stirring. Briefly, a mix of 9:1 volume ratio of H_2_SO_4_ and H_3_PO_4_ was stirred in an ice bath and potassium permanganate with graphite were slowly dropped into the solution under stirring for 30 min in either 85 °C or 95 °C. Then, 50 mL of deionized water was added and stirring was followed for either 30, 60 or 90 min. The solution was again placed in an ice bath and 20 mL of hydrogen peroxide with 150 mL of deionized water were added slowly to terminate the reaction. The mixture was left to cool down and finally, it was diluted with 20% HCl solution and it was centrifuged at 7500 rpm for 20 min. The liquid was decanted, and the precipitate was rinsed with deionized water so as to obtain a neutral pH. The synthesized GO samples were dried for 48 h at 80 °C in an oven. The demonstrated method produced GO with an O/C ratio of 0.8, in contrast to the conventional Hummers’ method that gives 0.44.

Reduced graphene oxide (rGO) is obtained by the chemical, electrochemical or thermal reduction of GO [36]. A thermal reduction method presented by Pei et al. [37] involves the swift heating of graphene oxide to allow the de-stacking and to obtain graphene by the rapid production of carbon monoxide and dioxide gases from the oxygen groups that are present in the surface of the sheets. A chemical reduction method would require a reducing agent to deoxygenate an exfoliated graphite oxide, such as hydrazine and dimethylhydrazine [38]. The electrochemical reduction can be achieved via the addition of graphite oxide in an aqueous colloidal suspension containing a buffer solution to produce rGO on the surface of an electrode. There are three ways of producing it, with a constant potential mode, with linear sweep voltammetry or cyclic voltammetry [39].

A common procedure of producing magnetic nanoparticles (MNPs) is with the hydrothermal method [40]. Haw et al. [41] presented a hydrothermal approach of precipitating ferrous sulfate and ferric chloride in a NaOH solution, with a molar ratio of OH^−^:Fe^2+^:Fe^3+^ at 8:1:2. The salts were added in a solution of 0.01 M aqueous HCl and nitrogen gas was used to prevent the formation of other iron oxides. The precipitate was added in an autoclave and placed at 200 °C in an oven for 1 h. The autoclave was left at room temperature to cool down and the black precipitate was rinsed with ethanol and deionized water. Lastly, the magnetic nanoparticles were dried at 45 °C for 24 h in an oven. Magnetic GO (mGO) can be obtained with the addition of 0.3 g of MNPs in an aqueous GO dispersion of 0.1% (0.3 g of GO in 300 mL deionized water) under ultrasonication for 30 min and then centrifuged and freeze-dried, as proposed by Kyzas et al. [42].

## 3. Bioanalytical Applications of Graphene Oxide for the Extraction of Small Organic Molecules

Until now, GO and its functionalized derivatives have been successfully coupled with various microextraction and miniaturized extraction sample preparation techniques. In this section the applications of graphene oxide derived materials in different sample preparation techniques for bioanalysis are discussed.

### 3.1. Solid-Phase Microextraction

Solid-phase microextraction (SPME) was introduced in the early 1990s by Pawliszyn et al. [43] to address the need for rapid and solvent-free extraction techniques. In SPME, extraction takes place at the outer coating of a thin fused-silica fiber coated with a layer of adsorbent. For the extraction, the extracting phase is exposed to the sample matrix either directly (direct immersion SPME) or at the headspace above the sample (headspace SPME) for a certain time span to reach equilibrium. Subsequently, the fiber is removed and desorption of the adsorbed analytes is performed by the addition of an appropriate solvent or thermally in the injection port of a gas chromatograph (GC) [44]. In SPME, the fiber coating plays a vital role in the extraction process, and the development of new coatings with high extraction efficiency is a significant research direction in this field [45]. This sample preparation technique exhibits a plethora of benefits including high sensitivity, small sample volume, rapidity and simplicity, while it can also be solvent-free [46].

Hajebi et al. [45] developed electrospun polyamide/GO/polypyrrole composite nanofibers for the headspace SPME of methamphetamine from urine samples prior to its determination by GC-MS. The composite fibers combined high surface-to-volume ratio and spun ability around a thin rod of the polyamide nanofibers with the great mechanical, thermal, and chemical stability and multifunctionality of GO/polypyrrole. Under optimum conditions, the LOD and the limits of quantification (LOQ) for the target analyte were 0.9 and 3.0 μg·L^−1^, respectively.

GO and functionalized GO derivatives have been also evaluated as stationary phases for in-tube SPME. In-tube SPME is an automated variation of conventional SPME that utilizes an open tubular capillary column as an extraction device [47]. This sample preparation technique offer various advantages in terms of automation, preconcentration and miniaturization [48]. Various GO composite materials have been evaluated as adsorbents for in-tube SPME in bioanalysis. Shamsayei et al. [49] developed a polythiophene/GO nanostructured electrodeposited coating for on-line electrochemically controlled in-tube SPME of amitriptyline and doxepin as antidepressant drugs prior to their determination by HPLC-UV. The composite coating was prepared on the inner surface of a stainless-steel tube by a facile in-situ electro-deposition method. In the composite coating, the GO acted as an anion dopant, as well as sorbent. Compared to polythiophene and GO coatings, the composite coating, exhibited long lifetime, good mechanical stability, as well as large specific surface area. A poly-ethylenedioxythiophene-GO electrodeposited coating fabricated on the inner surface of stainless steel tube has been also reported for the in-tube SPME of letrozole from plasma samples [50]. Also in this case, the GO acted both as anion dopant and as sorbent. Compared to pure poly-ethylenedioxythiophene coating, the combination of poly-ethylenedioxythiophene and GO created a more efficient sorbent the target analyte. Chen et al. [51] developed a GO/poly(3,4 ethylenedioxythiophene)/polypyrrole composite and used it as adsorbent for the in-tube SPME of 8-hydroxy-2′-deoxyguanosine, 3-hydroxyphenanthrene and 1-hydroxypyrene. The composite coating was electrodeposited on the internal surface of a stainless-steel tube and it exhibited good chemical and mechanical stability, high extraction efficiency, good resistance to matrix interference, as well as long lifespan.

Hollow-fiber SPME is another interesting alternative to conventional SPME, which eliminates the risk of cross-contamination and carry-over problems due to the utilization of disposable nature of the hollow fibers. Moreover, the hollow fibers can protect the sorbent, thus resulting in satisfactory stability and reliability of the extraction process [52]. Darvishnejad et al. [52] developed nanocubic cobalt oxide@GO nanocomposite reinforced hollow fibers for the hollow fiber-SPME of non-steroidal anti-inflammatory drugs from human urine prior to their determination by HPLC-UV. For this purpose, nanocubic Co_3_O_4_@GO was prepared through a hydrothermal method and dispersed in a solution in order to be immobilized in the wall pores of hollow fibers. Due to the synergistic effect between the Co_3_O_4_ nanocubes and the GO material, in combination with their unique three-dimensional interpenetrating porous network and high surface-to-volume ratio of GO, high extraction performance was reported.

Hyperbranched polyglycerol/graphene oxide nanocomposite has been employed for the reinforced hollow fiber solid/liquid phase microextraction of ibuprofen and naproxen from hair samples [53]. For this purpose, the surface of GO was modified with hyperbranched polyglycerol and the prepared nanocomposite was wetted by a few microliters of 1-octanol as organic solvent, followed by application for the extraction of the target analytes. The proposed method was found to be rapid, simple, sensitive and it required low solvent consumption.

### 3.2. Magnetic Solid-Phase Extraction

**Figure 1:**
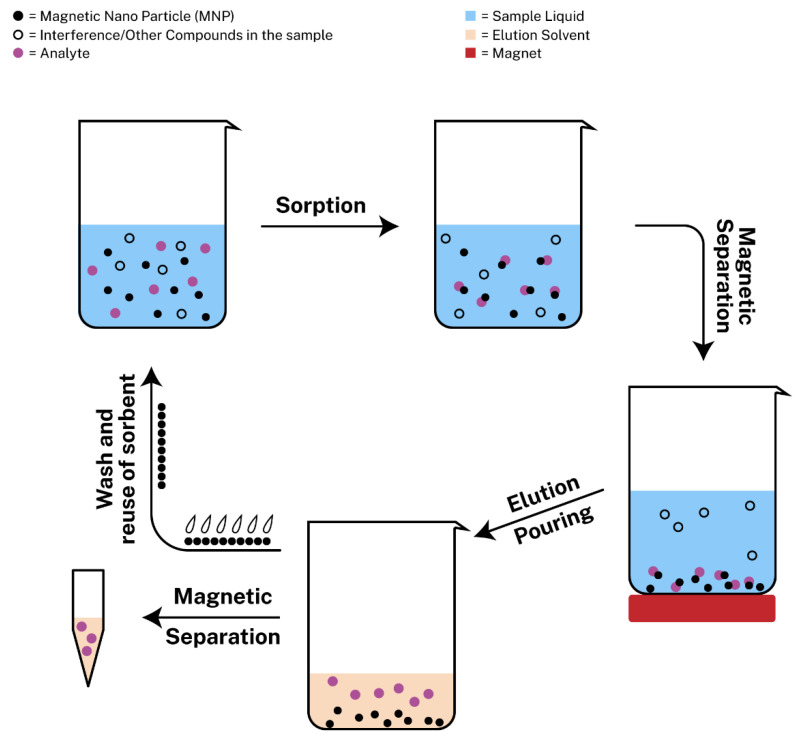
Schematic representation of magnetic solid phase extraction (MSPE).

Magnetic solid-phase extraction (MSPE) is new approach of SPE with the usage of magnetic nanoparticles as adsorbents that was introduced by Safarikova et al. [54] in 1999. Since then, many scientists experimented with magnetic nanoparticles to develop MSPE protocols. Among a plethora of magnetic sorbents, magnetic graphene oxide (mGO) has attracted the interest of many researchers, due to its large surface area and its delocalized π-π electron interactions so as to extract metal ions or aromatic compounds from different samples [55]. MSPE exhibits various benefits including rapid phase separation, during which the sorbent can be retrieved from the sample with ease with the assistance of an external magnetic field [56]. The procedure consists of the following steps: (i) the addition of the magnetic nanoparticles in the sample containing the target analyte, (ii) the sorption of the analyte onto the adsorbent, (iii) the magnetic separation with the use of a strong magnet so as to pour the liquid sample containing possible interferences or other compounds that are not of interest, (iv) the addition of the elution solvent for the dispersion of the analyte, (v) the magnetic separation of the sorbent from the eluent, which is ready for analysis and (vi) the wash and regeneration of the sorbent for possible reusage. The regeneration step is required between different extraction/desorption cycles and it is typically performed by treating the sorbent with an appropriate organic solvent and aqueous solution to avoid incomplete elution of the target analytes that could limit the applications of the material [19,57]. The steps can also be seen in Figure 1.

Poly(2-aminobenzothiazole)-coated mGO nanocomposite was prepared by Asgharinezhad et al. for the determination of anti-inflammatory drugs and non-steroidal drugs in urine samples by HPLC-DAD [58]. This new material demonstrated high extraction efficiencies for diclofenac, naproxen and ibuprofen, with recoveries ranging from 85.5–90.5% for the three target analytes and low relative standard deviations.

Cephalosporins in spiked human urine were extracted with the usage of mixed hemimicelles of mGO and ionic liquid and determined by HPLC-UV. The ionic liquid used in this work was 1-hexadecyl-3-methylmidazoliumbromide, which was added along with a buffer solution of phosphate at pH 7 and an amount of mGO in a centrifuge tube, following ultrasonication in order to form mixed hemimicelles. A range of parameters that had an effect on extraction were studied and under optimal conditions, the LODs of the cephalosporins were 0.6–1.9 ng·mL^−1^, while the RSDs were low and the extraction recoveries were satisfactory [59]. Another team of scientists, prepared mGO coated with deep eutectic solvents (DES) to preconcentrate and extract methadone from urine and human blood plasma samples prior to its determination by GC-MS and GC-FID. The DES that showed the greatest results was choline chloride with 5,6,7,8-tetrahydro-5,5,8,8-tetramethylnaphthalen-2-ol (TNO) at a molar ratio of 1:2 [60].

Taghvimi et al. [61] utilized magnetic nano-GO as a sorbent for pseudoephedrine in urine determined by HPLC-UV, which showed extraordinary results with recoveries being as close as 96% and satisfactory RSDs, that render the proposed method suitable for diagnostic clinics. In another study, the same adsorbent was used for the extraction of methamphetamine from urine, which exhibited once again high recoveries with the LOD being 30 ng·mL^−1^ [62].

Mitrazapine and its metabolites (8-hydroxy mirtazapine, *N*-desmethylmirtazapine) were determined in human urine samples by HPLC-UV, exploiting the excellent properties of mGO-polyanaline nanocomposite in their extraction by ultrasonic-assisted magnetic dispersive micro SPE (UA-Md-μSPE). A fixed amount of adsorbent was added into a test tube, along with triton X100 and deionized water. Then, to achieve dispersion the solution was sonicated and afterwards it was swiftly injected into another test tube containing the three compounds. The newly formed solution was shaken so as to adsorb the analytes and a strong magnet was placed to separate the particles from the sample, which was decanted. The adsorbed compounds were eluted under sonication with methanol and the eluate was injected into the HPLC for analysis. High preconcentration factors were demonstrated, with LODs ranging from 0.4–1.1 ng·mL^−1^ and RSDs lower than 10.1% [63].

A 3D-magnetic graphene for the extraction of carvedilol from human blood plasma samples was used by Sereshti et al. [64], followed by its determination via HPLC-UV. 3D Graphene was selected, because it shows higher adsorption capacity and surface area, while centrifugation or filtration are not needed. In an additional work, magnetic graphene was used for the M-dSPE of non-steroidal anti-inflammatory drugs (NSAIDs) from human urine and plasma samples, determined by UHPLC-PDA. The selected drugs were ketoprofen, naproxen, furprofen, fluribiprofen, diclofenac and fenbufen and their extraction yields from the MSPE were also high [65].

The preparation of molecular imprinted polymer based on magnetic chitosan-GO was conducted for the extraction of fluoxetine from urine samples by Barati et al. [66]. Recoveries of the proposed method were reported as high as 96%, with the preconcentration factor reaching 500 and the LOD 0.03 μg·L^−1^.

Tamsulosin hydrochloride was extracted and preconcentrated from human plasma samples with the use of superparamagnetic GO and determined by HPLC-UV. The sample preparation procedure that was used was magnetic dispersive SPE (M-dSPE) under ultrasonication, with each cycle having a duration of approximately 20 min. The method showed a LOD of 0.17 ng·mL^−1^, low RSDs ranging from 4.2–5.0% and good recoveries between 98.1–101.4% [67].

Zhang et al. [68] prepared a mGO/β-cyclodextrin adsorbent for the extraction of antiepileptic drugs in plasma samples followed by determination by HPLC-DAD. This new material was used for the MSPE of diazepam, phenytoin and carbamazepine, providing low LODs and RSDs, along with recoveries ranging between 78.49–100.93% for the three drugs.

In order to extract trace amounts of chloropheniramine from human plasma, Daryakenary et al. [69] prepared a mGO@polythione nanocomposite for the M-dSPE of the compound. Thionine was polymerized onto the mGO’s surface with an oxidative polymerization reaction. This led to high extraction yields of 87.9–96.4%, to the preconcentr-ation of 50 times, while each cycle of sample pretreatment lasted only 8 min. The same adsorbent was studied by Zeeb et al. [70] so as to extract duloxetine from human plasma, determined by HPLC. The necessary extraction time was reported at only 6 min, with RSD 3.9% and a LOD of 0.5 ng·mL^−1^.

The synthesis of third generation dendrimers covalently bonded to mGO nanosheets was carried out for its use in MSPE of certain serotonin reuptake inhibitors (sertraline, fluoxetine, citalopram and fluvoxamine) from human plasma so as to exploit the abundance of function groups, its biocompatibility to the compounds, as well as its vast surface area. The reported results demonstrate reusability of the sorbent up to 20 times, recoveries around 89% and LODs of 0.3–0.9 ng·mL^−1^ [57].

Hua et al. [71] prepared a GO-encapsulated magnetic Zr-metal organic framework (Zr-MOF) with high affinity to extract and determine by UPLC-HRMS the hematoporphyrin and hematoporphyrin monomethyl ether in human urine samples. Firstly, magnetic microspheres were fabricated by a solvothermal approach and then dispersed in ethanol under sonication, followed by the dropwise addition of the solution into oleinic acid. Afterwards, the magnetic Zr-MOF, modified with *N-*(*n*-propyl)ethylenediamine, was synthesized by the addition of terephthalic acid and zirconium(IV) chloride into the Fe_3_O_4_-COOH dispersion and heated for half a day. After rinsing with ethanol and drying, the obtain solid is added in a solution of 95% v/v aqueous ethanol under ultrasonication and then *N-*(*n*-propyl)-ethylenediamine is dropped gradually. Lastly, the modified magnetic Zr-MOF was placed in a solution that consisted of a dispersion of GO in water along with certain amounts of *N*-ethyl-*N*-(3-(dimethylamino)propyl) carbodiimide and *N*-hydroxysuccinimide and amidation reaction was conducted. Pourbahman et al. [72] synthesized another type of mGO-MOF, the mGO-MOF-74 with polytyramine as a sorbent for the monitoring of trace amounts of prokinetic drugs (itopride and domperidone) in human plasma, thus achieving low LODs and RSDs.

Atorvastatin and simvastatin were determined by HPLC after their M-μSPE from urine with a sorbent compositing of mGO and a zeolite imidazolate framework-8 that were hybridized. Preconcentration factors of 169–191 were achieved, with LODs as low as 116 and 387 pg·mL^−1^ and high recoveries of 84.7–95.7% [73].

The preconcentration of codeine and morphine was achieved by using a m-rGO@Ag nanocomposite from human blood and urine samples. This nanocomposite was prepared by adding GO in deionized water, along with ferrous and ferric chloride salts to fabricate mGO and lastly silver nitrate was added into the solution under stirring. To reduce the GO, NaBH_4_ was used and slowly added into the mixture [74]. The same group of scientists also experimented with mGO@di-(2-ethylhexyl) phosphoric acid for the extraction of propyl paraben, methyl paraben, phenol and bisphenol A from urine and breast milk samples prior to their HPLC-UV determination [75].

Additional application of mGO in MSPE regards the extraction of psychoactive drugs (amphetamine, 6-monoacetylmorphine, morphine, methamphetamine, cocaine, codeine, benzoylecgonine and dolantin) in urine samples that were determined with UHPLC-MS/MS [76].

Yuvali et al. [77] used a green approach of preparing a magnetic carbon nanodot/GO hybrid adsorbent for the extraction of ibuprofen in human plasma samples, determined by HPLC-DAD. This new material exhibited extraction yields of 91–95%, with RSDs lower than 4%. The proposed method showed a limit of detection of 8 ng·mL^−1^ [77]. Table 1 presents the applications of MSPE for bioanalysis with GO-based nanomaterials.

### 3.3. Stir Bar Sorptive Extraction

Stir bar sorptive extraction (SBSE) is a sample preparation technique that was initially introduced by Baltussen et al. in 1999 [78]. Since its introduction, SBSE has attracted the attention of many analytical scientists and currently it is widely applied for the extraction/preconcentration of non-polar analytes from various matrices. Some benefits of SBSE are its simplicity, the reduced organic solvent consumption and the high sensitivity [79]. In SBSE, extraction takes place by direct immersion or headspace exposure of a stir bar that incorporates a magnet core and it is typically composed of a PDMS film, coated onto a glass jacket [80]. Most applications of SBSE utilize a PDMS coated stir bar and although polyethylene glycol and polyacrylate stir bars are currently available, the applicability of SBSE to more polar and hydrophilic target analytes is limited. As a result, the development of more selected SBSE media is in the forefront of research to expand the applicability of this extraction technique [80]. Common approaches for the preparation of in-house coatings for SBSE media include the sol-gel technology, the synthesis of monolithic materials, the adhesion technique, the molecular imprinting technique, and the solvent exchange procedures [80,81].

A nano graphene oxide sol-gel (NGO/sol-gel) composite coating stir bar was prepared using methyltrimethoxysilane and tetraethoxysilane as sol-gel precursor, through deposition on the surface of a capillary glass tube. The novel bars were used for extraction of potent central nervous system stimulants from urine samples prior to their determination by HPLC-UV [82]. Rapid extraction and high selectivity were reported, as well as low consumption of organic solvents. The LOD values of the proposed method were 10 and 11 ng·mL^−1^ for methamphetamine and amphetamine, respectively.

Fan et al. [83] developed a water-compatible GO/MIP coated stir bar for the extraction of propranolol from urine samples followed by its determination by HPLC-UV. For this purpose, in situ polymerization was employed for linking the composite coating to the stir bar. The novel stir bars exhibited good mechanical and chemical stability, good recognition ability in aqueous samples and good adsorption capacity for propranolol. The enrichment factor for propranolol was 59.7 and the extraction recovery was 59.7%.

GO-silica composite reinforced hollow fibers have been proposed as a device for pseudo-stir bar solid phase microextraction of sulfadiazine from urine samples prior to its spectrophotometric determination. For the fabrication of the hollow fibers, GO-silica sol was withdrawn with a syringe, injected into the lumen of the fiber and maintained for 30 min to fully evaporate the solvent. Cetyltrimethylammonium bromide was added to the sol solution as surfactant to increase the absorbance of sulfadiazine. After three repetitions of this procedure, the modified fibers were dried and used as microextraction devices. The proposed bars were simple and cost-effective, while it demonstrated good performance characteristics [84].

Hollow fiber coated Fe_3_O_4_@maleamic acid-functionalized GO has been employed for the SBSE of ibuprofen, aspirin, and venlafaxine from human urine samples prior to their determination by GC-MS [85]. For this purpose, the novel sorbent was prepared and ultrasonically penetrated in the outer surface pores of the hollow fiber in the presence of a nonionic surfactant (Triton X-140). Compared to magnetic graphite, magnetic graphene and magnetic multiwalled carbon nanotube, the magnetic graphene oxide provided higher recoveries for all analytes. The functionalization of the GO was maleamic acid was performed to increase the selectivity of the sorbent. Moreover, the functionalization of the sorbent with magnetite, helped to rotate the hollow fiber in the magnetic field. The proposed methodology was rapid, cost-effective, while it provided high recovery, and no memory effect with good recoveries.

### 3.4. Pipette Tip Solid-Phase Extraction

Pipette tip solid-phase extraction (PT-SPE) is a sample preparation technique that was developed to reduce the extraction time and organic solvent consumption compared to traditional SPE methodologies. In a typical PT-SPE setup, a sorbent is dispersed into a pipette tip and the extraction of the target analytes is performed by passing through the tip, based on the same principles as in SPE. As such, the sorbent is initially activated by an appropriate solvent or a combination of solvents to enable the appropriate molecular interaction the sorbent and the target analyte. Subsequently, the sample is aspirated into the PT-SPE device and the analytes are extracted, followed by washing of the sorbent to remove interferents and elution of the target analytes by aspirating an appropriate elution solvent several times to rupture the interaction between the target analytes and the sorbent. PT-SPE offers multiple advantages, such as simplicity, high efficiency, as well as reduced consumption of sample and organic solvents [86,87].

Reduced GO (RGO) has been used to prepare a PT-SPE device for the extraction of indometacin and acemetacin from human urine [88]. For this purpose, the sorbent was placed in a pipette tip cartridge, while cotton was placed in the ends of the tip to avoid sorbent loss. The extraction efficiency of the PT-SPE device was compared to the extraction efficiency of conventional SPE cartridges that contained the same amount of RGO. Higher extraction recoveries were observed with the PT-SPE device, since higher sorbent bed was created in this case. Moreover, the novel method exhibited higher overall extraction efficiency in comparison with commercially available SPE cartridges and other sorbents (e.g., MWCNTs).

The functionalization of GO with ionic liquids and deep eutectic solvents to develop PT-SPE extraction methods has been also investigated. Zhang et al. [19] prepared a three-dimensional ionic liquid-ferrite functionalized GO nanocomposite and used it for the PT-SPE of 16 polycyclic aromatic hydrocarbons from human blood samples. The miniaturized extraction method exhibited lower organic solvent and sample consumption compared to conventional SPE, while the PT-SPE device was found to be reusable for at least 10 times. Under optimum conditions, extraction of the target analytes was completed in 10 repeated aspirating/dispensing cycles, while elution was performed in 5 repeated aspirating/dispensing cycles. Due to the high specific surface area of the nanocomposite and its ability to adsorb PAHs by π-stacking interaction, better performance was observed compared to C_18_ and MWCNT sorbents.

Yuan et al. [89] prepared a deep eutectic solvent functionalized GO composite adsorbent through reversible-addition fragmentation chain-transfer polymerization and used it for the PT-SPE of toluene and xylene exposure biomarkers from urine samples prior to their determination by HPLC-UV. Allyltriethylammonium bromide/ethylene glycol was employed for the covalent modification of the sorbent. The novel sorbent enabled multiple adsorption interactions with the target analytes, which included π-interaction, hydrogen bonding, electrostatic adsorption, and hydrophobic interaction. Thus, good adsorption ability was observed.

### 3.5. Other Extraction Techniques

Porphyrin-functionalized GO sheets have been employed for the micro SPE (μSPE) of non-steroidal anti-inflammatory drugs from urine samples [90]. The composite sorbent combined the benefits of GO (i.e., its high specific surface area) with the benefits of porphyrins (i.e., structural characteristics and tunability of their terminal functional groups). The novel sorbent was packed into a μSPE cartridge which was found to be reusable for more than 50 times. The LODs for the target analytes were in the range of 0.5 to 2.0 ng·mL^−1^. The overall method was cost-effective and simple, as well as good sample cleanup.

Sereshti et al. [91] prepared an electrospun polyethylene terephthalate/GO nanofibrous membrane for the extraction of tamoxifen from human blood plasma samples. The membrane was in circular shape and fixed in a filter holder that was further attached to a syringe. Extraction of the target analyte took place by placing the sample solution in the syringe and passing through the filter. Good analytical performance was observed, as well as sorbent reusability.

GO derivatives have been utilized as adsorbents for microextraction by packed sorbents (MEPs) [92]. In MEPs, the sorbent is packed inside a micro-syringe in different positions, such as the barrel or the needle. In this technique, the sample is drawn through a syringe and the analytes are extracted by passing through the sorbent. Subsequently, the sorbent is then washed to remove interfering compounds from the sample and the analytes are eluted by an appropriate solvent [93]. Ahmadi et al. [92] used RGO as a sorbent to develop a MEPs method coupled with HPLC-MS determination for the quantification of local anesthetics in human plasma and saliva samples. The utilization of RGO as the sorbent for MEPS provided good extraction capacity and selectivity towards the target analytes.

Fabric phase sorptive extraction (FPSE) is another sample preparation technique that has been employed for the determination of antidepressant residues in human blood serum prior their determination by HPLC-DAD [94]. FPSE is a sample preparation technique that was invented by Kabir and Furton in 2014 and since then it has attracted the interest of many researchers working in the field of sample preparation. In FPSE, the sorbent is covalently bonded to a substrate surface, providing high chemical, solvent, and thermal stability. Due to the open geometry of FPSE, fast analyte adsorption and desorption can be achieved [95,96]. In the methodology proposed by Lioupi et al. [94], sol-gel graphene sorbent, coated on cellulose FPSE media was employed for the extraction of the drugs. The developed method could simplify the sample preparation workflow, while it eliminated the need for protein precipitation of biological samples prior to the extraction.

Thin film microextraction (TFME) utilizing GO as adsorbent has been also employed for the determination of small organic molecules from biological samples. TFME was introduced in 2003 by Pawliszyn et al. [97] in order to address the limiting uptake rate and capacity sometimes observed with SPME [98]. TFME is characterized by enhanced volume and the surface-to-volume ratio of the extraction phase, thus higher extraction rate and shorter extraction time can be achieved compared to conventional SPME methodologies. Ghani et al. [99] developed magnesium-aluminum-layered double hydroxide-GO composite mixed-matrix membranes for the thin-film microextraction of diclofenac from urine and plasma samples. In this case, the composite was incorporated into a mechanically stable polyvinylidene difluoride membrane. High extraction efficiency and reproducibility was observed, as well as good stability and reusability. The same research group fabricated a woven cotton yarn-GO-layered double hydroxide (GO-LDH) composite as a sorbent for the TFME of non-steroidal anti-inflammatory drugs from urine and plasma samples [98]. In this case, the substrate was woven and employed as the substrate for the construction of GO layers, to improve the contact area between the sorbent and the target analytes. Moreover, the contribution of broad surface of GO flakes for the accommodation of the layered double hydroxide and the preparation of GO-LDH composites enhanced the extraction efficiency of the sorbent.

Zohdi et al. [100] developed thin layer GO tablets by utilizing a mixture of GO and polyethylene glycol on a polyethylene substrate for the sample preparation of biological fluids. The developed tablets were used for the determination of omeprazole in human saliva for liquid chromatography tandem mass spectrometry. Extraction of the target analytes was performed by immersing the tablets into the sample solution. Subsequently, the tables with the adsorbed analytes were removed and elution took place by immersing the tablet in acetonitrile. The proposed method was simple and rapid and the proposed tablets were reusable for at least 10 times.

GO has been also employed in electromembrane extraction (EME) of small organic molecules from biological samples. EME is a recently introduced sample preparation technique in which an electrical potential is applied to facilitate the extraction of analytes across a hollow fiber membrane. The hollow fiber is filled with an organic solvent (acceptor phase) and it separates the sample solution (donor phase) from the acceptor phase. As a result, charged analytes can be drawn across the liquid membrane towards the organic solvent by the implementation of a potential. Among the benefits of EME is its simplicity, rapidity, cost-effectiveness and usage of small quantities of organic solvents [101]. Bagheri et al. [101] proposed the GO assisted electromembrane extraction of methamphetamine from hair and urine samples. It was observed that the presence of GO in the hollow fiber membrane wall increased the effective surface area, the strength of interactions with the analyte and the polarity of support liquid membrane, thus resulting in enhanced migration of the target analyte. In conclusion, the immobilization of GO in membranes is an excellent way to enhance the performance of EME technique.

## 4. Conclusions

Unequivocally, GO and its functionalized derivatives are efficient sorbents for the extraction of small organic molecules from complex biological samples. Within the ultimate goals upon the development of novel materials for various applications is to achieve the desired features towards the targeted application bearing in mind the modern trends of sustainability. A strategy to achieve so is to avoid the metal- or polymer-based materials. Graphite, as well as its chemical modified derivatives are considered to be green oriented materials. These materials have been successfully coupled with a plethora of green analytical sample preparation techniques (e.g., SPME, SBSE, PT-SPE, FPSE, d-SPE, MSPE etc.), proving that their use in different formats of SPE methods is possible. As such, these materials enrich the toolbox of scientists working in the field of bioanalysis. The selection of the material can be based on the need for the selectivity and sensitivity of the determination, while the selection of the sample preparation technique can be also based on the equipment availability and analysis requirements. It should be also mentioned that the GO-based materials were found to be reusable for multiple extractions in many cases, after an appropriate regeneration step between the different extraction/desorption cycles. Additionally, even the used GO-based materials could be potentially used either directly for alternative applications or as additives for other advantageous materials/composites.

Future perspectives in this field include the development of more functionalized GO derivatives, which could selectively extract the target analytes from the complex matrix. The combination of GO-based sorbents with other powerful materials, such as molecularly imprinted polymers (MIPs) and metal-organic frameworks (MOFs) to create sorbents with enhanced selectivity and high surface area is also a promising trend for sorbent development. The exploration of more sample preparation techniques (e.g., capsule phase microextraction) that have gained attention lately due to their compliance with the requirements of GAC is also a future perspective of the bioanalytical applications of graphene oxide for the extraction of small organic molecules. In addition, attention should be also paid to the development of automated techniques that can increase the accuracy and the reproducibility of the determination and decrease the sample consumption and the usage of organic solvents. Finally, it should be noted that most of the developed methodologies are used for the analysis of blood serum, blood plasma and urine samples. The applications of GO sorbents and their functionalized derivatives could expand to the analysis of alternative biological samples (e.g., saliva, oral fluid, sweat etc.), which are also a valuable source of information.

## Figures and Tables

**Table 1:** Recent applications of graphene-based nanomaterials in bioanalysis.

Adsorbent	Analyte(s)	Applications	LODs (ng·mL^−1^)	EF ^1^	Reference
Poly(2-aminobenzothiazole)-mGO	NSAIDs	Urine	0.07–0.3	35.7–37.7	[58]
mGO-IL	Cephalosporins	Spiked urine	0.6–1.9	4	[59]
mGO-DES	Methadone	Urine, plasma	2.5–14.3 × 10^−3^	500	[60]
Nano mGO	Pseudoephedrine	Urine	25	NA ^2^	[61]
Nano mGO	Methamphetamine	Urine	30	168	[62]
mGO-polyaniline	Mirtazapine, 8-hydroxy mirtazapine, *N*-desmethyl mirtazapine	Urine	0.4–1.1	158, 124, 109	[63]
3D-m Graphene	Carvedilol	Plasma	0.5	NA	[64]
3D-m Graphene	NSAIDs	Urine, plasma	0.61–1.2	10	[65]
GO-magnetic chitosan	Fluoxetine	Urine	0.03	500	[66]
Superpara-mGO	Tamsulosin hydrochloride	Plasma	0.17	10	[67]
mGO-β-cyclodextrine	Antiepileptic drugs	Plasma	11.89–47.1	NA	[68]
mGO-polythione	Chloropheniramine	Plasma	0.4	16.7–18.3	[69]
mGO-polythione	Duloxetine	Plasma	0.5	NA	[70]
mGO-dendrimer	Serotonin reuptake inhibitors	Plasma	0.3–0.9	30	[57]
mGO-Zr-MOF	Hematoporphyrin, hematoporphyrin monomethyl ether	Urine	3.6, 4.2	NA	[71]
mGO-MOF-74	Prokinetic drugs	Plasma	0.4, 1.1	18, 17.6	[72]
mGO-ZIF-8	Atorvastatin, simvastatin	Urine	116 × 10^−3^, 387 × 10^−3^	169.4–191.4	[73]
m-rGO-Ag	Codeine, morphine	Blood, urine	1.8 × 10^−3^, 2.1 × 10^−3^	1000	[74]
mGO-di-(2-ethylhexyl) phosphoric acid	Methyl & propyl paraben, phenol, bisphenol A	Breast milk, urine	2.5–14.3 × 10^−3^	500	[75]
mGO	Psychoactive drugs	Urine	0.02–0.2	NA	[76]
mGO-carbon nanodot	Ibuprofen	Plasma	8	7.5	[77]

^1^ EF = Enrichment Factor. ^2^ NA = Not Available.
